# Application of laboratory frailty index in predicting delirium in elderly patients with community-acquired pneumonia

**DOI:** 10.3389/fragi.2024.1478355

**Published:** 2024-12-16

**Authors:** Jingxian Liao, Xiaozhu Shen, Zhiqiang Du, Lei Miao

**Affiliations:** ^1^ Department of Geriatrics, The Second People’s Hospital of Lianyungang, Lianyungang, Jiangsu, China; ^2^ Department of Critical Care Medicine, The Second People’s Hospital of Lianyungang, Affiliated Hospital of Kangda College of Nanjing Medical University, Lianyungang, Jiangsu, China

**Keywords:** elderly, community-acquired pneumonia, malnutrition, frailty, delirium

## Abstract

**Background:**

With the global aging population, community-acquired pneumonia and delirium are increasingly critical health issues among the elderly. The Laboratory Frailty Index provides an objective measure of frailty. This study explores its capacity in predicting delirium and examines the interplay between frailty and nutritional status in elderly patients with community-acquired pneumonia.

**Methods and materials:**

This retrospective study included 481 elderly patients aged 75 and above diagnosed with community-acquired pneumonia. The Laboratory Frailty Index was calculated by dividing the sum of abnormal indicator scores by the total number of test indicators, resulting in a score ranging from 0 to 1, with higher values indicating greater frailty.

**Results:**

Higher Laboratory Frailty Index scores were associated with an increased risk of delirium. The index’s predictive accuracy improved when combined with nutritional assessments. Patients experiencing malnutrition alongside higher frailty scores exhibited a higher risk of adverse outcomes. Nutritional status mediated the relationship between frailty and delirium, underlining the significance of addressing both variables.

**Conclusion:**

The Laboratory Frailty Index is a robust predictor of delirium in elderly patients with community-acquired pneumonia. These findings provide valuable insights for the early identification and intervention of delirium in clinical settings.

## Highlights

This study pioneers the use of FI-Lab for predicting delirium in elderly CAP patients.
Combining FI-Lab with MNA-SF improves prediction accuracy for delirium.
It uncovers the mediating role of MNA-SF between FI-Lab and delirium, underscoring the interaction between frailty and nutritional status.

## Introduction

As the global population ages, health issues among older adults are receiving increasing attention. Community-acquired pneumonia (CAP) is prevalent in this demographic, leading to high hospitalization and mortality rates (Torres et al., 2019). Delirium, an acute brain dysfunction, is particularly common among hospitalized elderly patients, with incidence rates between 30%–50% (Marcantonio, 2017). It prolongs hospital stays, raises medical costs, increases mortality rates, and results in long-term functional impairments (Inouye et al., 2014). Early identification and intervention are crucial (Soysal et al., 2019), yet pose significant challenges in geriatric care.

Frailty has emerged as a key indicator of health status in older adults, strongly linked to adverse outcomes such as falls, hospitalizations, and mortality (Parrotta et al., 2023). It is also an independent risk factor for delirium (Dani et al., 2018). Traditional frailty assessment tools, including the Fried Frailty Phenotype (Sanchez et al., 2020), the Frailty Index (based on deficit accumulation) (Blodgett et al., 2017), and the Clinical Frailty Scale (CFS) (Rockwood et al., 2015), rely on clinical questionnaires and physical examinations. While informative, these methods have limitations: (1) Subjectivity: Assessments can be influenced by the evaluator’s experience, leading to inconsistency (Ide et al., 2021); (2) Time-Consuming: Comprehensive assessments are often impractical in acute settings where rapid decision-making is needed. (3) Limited Predictive Ability for Delirium: Existing tools may not fully capture the complex physiological changes associated with delirium risk (Fan et al., 2020).

The Laboratory Frailty Index (FI-Lab) has emerged as a novel approach to frailty assessment. Unlike traditional tools, FI-Lab is based on routinely collected laboratory test results, providing an objective and quantifiable measure of physiological reserve and health status (Wang et al., 2022). By synthesizing abnormalities across multiple laboratory parameters, FI-Lab reduces subjectivity and integrates easily into clinical workflows (Sanchez et al., 2020). FI-Lab offers several advantages: (1) Objectivity: Minimizes evaluator bias by using objective laboratory data; (2) Efficiency: Utilizes existing laboratory results without requiring additional assessments, making it suitable for acute care settings; (3) Comprehensive Health Indicator: Reflects systemic physiological dysfunctions that may not be apparent in physical assessments alone (Resendes et al., 2023).

Despite its potential, the role of FI-Lab in predicting delirium among elderly patients remains underexplored. Delirium is a multifactorial condition influenced by various physiological disturbances (Sahle et al., 2022). Traditional frailty indices may not adequately predict delirium risk due to their focus on physical or functional deficits without capturing underlying biochemical abnormalities (Rockwood and Mitnitski, 2007). Delirium is associated with metabolic imbalances and inflammatory processes not assessed by conventional frailty tools. Patients identified as non-frail by traditional tools may still be at high risk of delirium due to unseen physiological vulnerabilities. Therefore, exploring FI-Lab’s predictive role for delirium could address these gaps by incorporating laboratory markers indicative of physiological stress and organ dysfunction (Guan et al., 2022; Ellis et al., 2020).

This study aimed to investigate the predictive capacity of FI-Lab for delirium in elderly patients with CAP. By examining the interaction between frailty and nutritional status, we sought to determine whether FI-Lab can serve as an effective tool for early identification of patients at high risk of delirium, thereby informing interventions to improve outcomes.

## Methods

### Study design and patients

This retrospective study used purposive sampling to select 481 elderly patients (aged ≥75 years) with community-acquired pneumonia (CAP) admitted to the Second People’s Hospital of Lianyungang between June 2020 and June 2024.

### Inclusion criteria

1. Diagnosis of CAP based on Infectious Diseases Society of America/American Thoracic Society (IDSA/ATS) guidelines (Metlay et al., 2019).
2. Age ≥75 years.
3. Hospitalization for >48 h to allow sufficient time for delirium observation.
4. Complete laboratory test data and clinical records.
5. Inclusion of both male and female patients.

### Exclusion criteria

1. Records of delirium within 30 days prior to admission.
2. Presence of severe neurological disorders (e.g., Parkinson’s disease, Alzheimer’s disease) or mental illnesses (e.g., schizophrenia, major depression).
3. Patients in the terminal stages of malignant tumors or severe infections who could not participate in the study.
4. Missing laboratory test data or clinical records.
5. For patients hospitalized multiple times for CAP during the study period, only the first hospitalization records were included; subsequent records were excluded.

This study did not involve direct interventions in laboratory tests or clinical treatments. Ethical approval was obtained from the Ethics Committee of the Second People’s Hospital of Lianyungang (No. 2022K040), and the study adhered to the Declaration of Helsinki. Informed consent was waived due to the retrospective nature of the study.

### Data collection

Clinical and laboratory data were extracted from the hospital’s electronic medical record system. Collected data included demographic characteristics, underlying diseases, admission and discharge details, discharge outcomes, and clinical results. Laboratory data focused on routine indicators obtained within the first 24 h of admission, including: (1) Complete blood count: Hemoglobin (Hb), white blood cell count (WBC), platelet count (PLT). (2) Biochemical tests: Liver function (ALT, AST), kidney function (BUN, SCr), glucose (Glu), albumin (Alb), triglycerides (TG), total cholesterol (TC). (3) Inflammatory markers: C-reactive protein (CRP).

### Nutritional assessment

The Mini Nutritional Assessment-Short Form (MNA-SF) was used to evaluate nutritional status (Liu et al., 2022). It comprises six questions that collectively generate a score assessing nutritional status, addressing aspects such as appetite loss, weight changes over the past 3 months, mobility, psychological stress or acute illness, neuropsychological problems, and Body Mass Index (BMI). Scores range from 0 to 14, categorizing nutritional status as: normal (12–14 points), at risk of malnutrition (8–11 points), and malnutrition (0–7 points).

### Delirium assessment

Delirium was assessed using the Confusion Assessment Method (CAM) (Green et al., 2019). The CAM focuses on four core features: (1) acute onset and fluctuating course, (2) inattention, (3) disorganized thinking, and (4) altered level of consciousness. Diagnosing delirium requires the presence of both Feature one and Feature 2, plus either Feature 3 or Feature 4.

### Definition and outcome

The FI-Lab quantifies frailty by assessing abnormalities in routine laboratory and vital sign indicators (Metlay et al., 2019; Blodgett et al., 2016). Blood pressure, a critical marker of cardiovascular health, when abnormal may indicate risks such as hypertension or hypotension, thereby affecting both the overall health and functional status of older individuals. Similarly, heart rate is a direct indicator of cardiac function; deviations from the norm may suggest arrhythmias or heart diseases impacting the quality of life in the elderly (Williams et al., 2018). The WBC count reflects immune system efficiency, where abnormalities could point to infections, inflammation, or hematological disorders—particularly significant for older adults with potentially compromised immunity. Hemoglobin levels illustrate the blood’s oxygen-carrying capacity, with aberrations potentially indicating anemia, malnutrition, or chronic diseases. Platelet levels are indispensable for coagulation, and irregularities may denote coagulation disorders (Patel, 2008). ALT and AST are hepatic function markers; abnormal values suggest liver damage or diseases like hepatitis or cirrhosis. Lipid profile indicators, total cholesterol and triglycerides, when abnormal, may highlight risks for cardiovascular diseases such as atherosclerosis. Blood glucose levels are vital markers for diabetes and metabolic syndrome, with deviations suggesting insulin resistance or diabetes. Renal function is evaluated through creatinine and BUN levels; abnormalities may signal renal dysfunction. Albumin levels indicate nutritional and liver status, where low values could suggest malnutrition, chronic illness, or hepatic dysfunction (Moreno et al., 2019). CRP serves as an inflammation and infection marker, with elevated levels pointing to acute or chronic inflammatory conditions (Ridker, 2014). The integration of these indicators allows for a comprehensive health evaluation, assisting in the identification of potential health risks and functional decline within the elderly demographic. Consequently, the FI-Lab provides clinicians with an objective metric for assessing health status and frailty in elderly patients. In our study, we selected 15 variables comprising three vital signs and 12 laboratory indicators. The normal reference ranges are as follows: SBP: 90–140 mmHg; DBP: 60–90 mmHg; Heart rate: 60–99 bpm; WBC count: 4.0–11.0 × 10⁹ cells/L; Hemoglobin: Males: 12–16 g/dL, Females: 11–15 g/dL; Platelet count: 100 - 300 × 10⁹ cells/L; ALT: 7–56 IU/L; AST: 10–40 IU/L; TC: <200 mg/dL; TG: <150 mg/dL; Glu: 70–110 mg/dL; Scr: Males: 0.74–1.35 mg/dL, Females: 0.59–1.04 mg/dL; BUN: 6–20 mg/dL; ALB: 3.5–5 g/dL; CRP: 0.42–5.2 mg/L. Values outside these ranges were scored as 1, while normal values were scored as 0. The FI-Lab index was calculated as the ratio of abnormal indicators to the total number of indicators assessed. For example, if three out of ten indicators were abnormal, the FI-Lab score would be 0.3, indicating moderate frailty. Scores range from 0 to 1, with higher values reflecting greater frailty.

### Statistical analysis

Statistical analyses were performed using IBM SPSS Statistics version 21 and R 4.4.1. All statistical tests were two-sided, with a significance level set at *p* < 0.05.

### Handling of missing data

To ensure the robustness of the analyses, missing data were addressed using Multiple Imputation by Chained Equations (MICE). This method accounts for uncertainty associated with missing data and provides unbiased parameter estimates. Imputation was conducted in R using the mice package with five iterations to stabilize imputed values. Variables with more than 20% missing data were excluded to prevent potential bias.

### Descriptive statistics

Descriptive statistics were used to summarize the demographic and clinical characteristics of the study population. Continuous variables were expressed as mean ± standard deviation (SD) or median (interquartile range, IQR), depending on the normality of their distribution. Categorical variables were presented as frequencies and percentages.

### Comparative analysis

For continuous data following a normal distribution, the Student’s t-test was applied to compare means between two groups. For non-normally distributed data, the Mann-Whitney U test was used to compare medians. Chi-square tests were employed to examine associations between categorical variables.

### Logistic regression

To assess the impact of FI-Lab and MNA-SF scores on delirium, logistic regression analyses were conducted in three models: Model one without adjustments, Model two adjusted for age, gender, and Charlson Comorbidity Index (CCI), and Model 3 further adjusted for additional variables, including vital signs, as well as laboratory indicators such as white blood cell count, hemoglobin, albumin, blood urea nitrogen, glucose, and C-reactive protein. Odds ratios (OR) and 95% confidence intervals (CI) were calculated for each model.

### ROC curve analysis

Receiver operating characteristic (ROC) curves and the area under the ROC curve (AUC) were calculated to assess the predictive value of the FI-Lab, MNA-SF, and their combined detection. Patients were categorized into two groups based on the cut-off value of the FI-Lab. The cut-off value for the FI-Lab was determined through ROC curve analysis, selecting the value that maximized both sensitivity and specificity.

### Survival analysis

Kaplan-Meier survival analysis was conducted to evaluate the impact of the FI-Lab on survival outcomes in patients with different nutritional statuses. A log-rank test was used to determine whether there was a statistically significant difference in survival between these groups.

### Mediation analysis

Using the Bootstrap approach with 5,000 repeated samples, we assessed the mediating effect of the MNA-SF and FI-Lab. The mediating effect was considered statistically significant if the 95% confidence interval did not include zero.

## Results

### Patient characteristics

**FIGURE 1:**
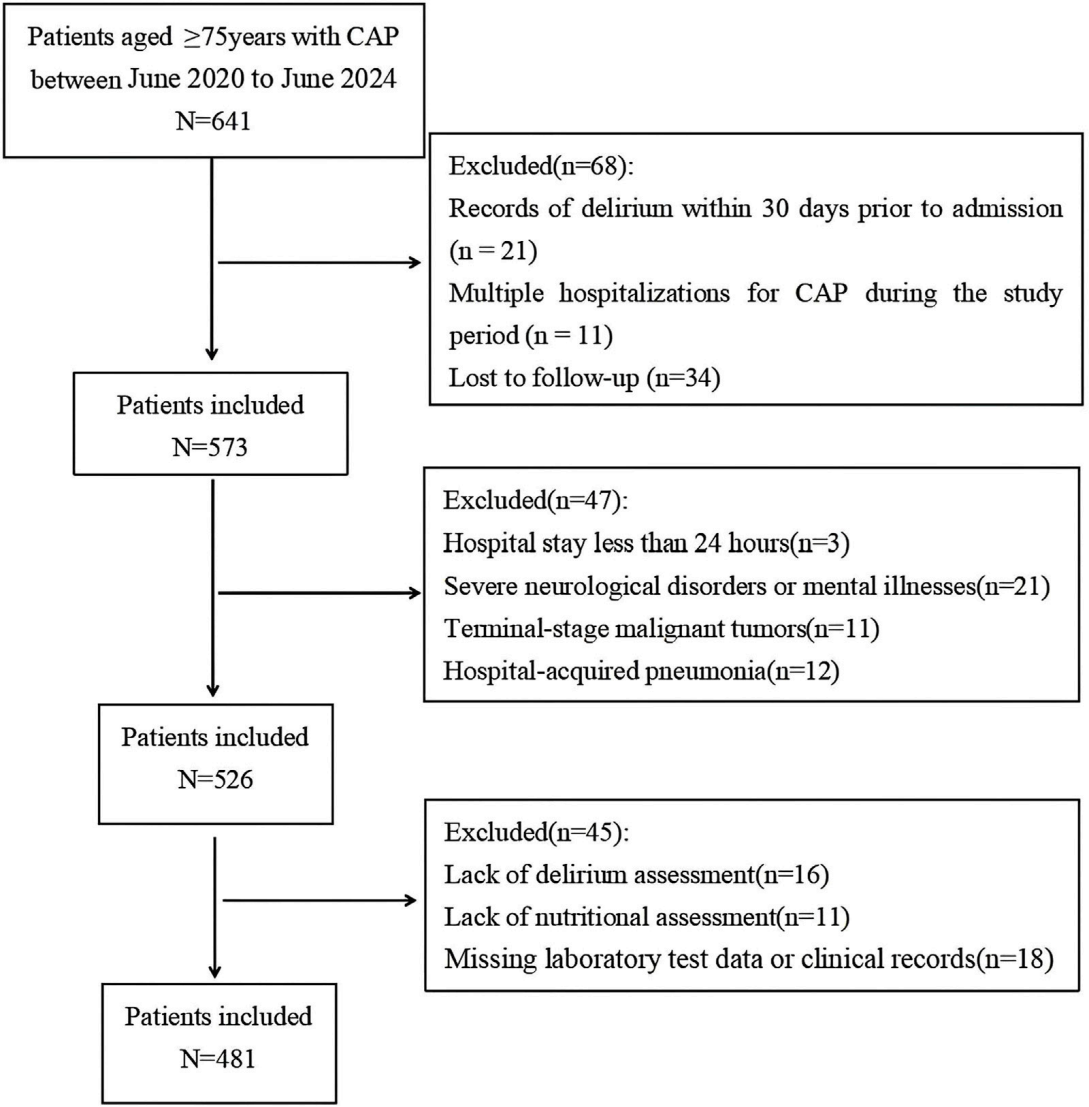
Flow diagram displaying the progress of all participants through the study.

The study enrolled 481 elderly CAP patients, adhering to specific inclusion and exclusion criteria as outlined in Figure 1. The participants had an average age of 84.39 ± 5.14 years, with a delirium incidence rate of 21.4% and a 28-day mortality rate of 9.6%. Comparative analysis revealed that the delirium group had significantly higher values than the non-delirium group in terms of age (P< 0.001), mortality rate (P< 0.001), incidence of SCAP (P< 0.001), CCI score (P< 0.001), malnutrition (P< 0.001), polypharmacy (*p* = 0.018), blood glucose (P< 0.001), total cholesterol (*p* = 0.027), triglycerides (*p* = 0.004), blood urea nitrogen (*p* = 0.002), serum albumin (P< 0.001), hemoglobin (*p* = 0.002), C-reactive protein (P< 0.001), and FI-Lab (P< 0.001) (Table 1).

**TABLE 1:** Baseline characteristics of elderly CAP patients.

Variables	Total (n = 481)	Non-delirium group (n = 378)	Delirium group (n = 103)	P
Gender				0.434
Male, n (%)	266(55.3)	213(56.3)	53(51.5)	
Female, n (%)	215(44.7)	165(43.7)	50(48.5)	
Age, mean (SD)	84.39(5.14)	83.86(4.73)	86.34(6.07)	<0.001
Outcome				<0.001
Survival, n (%)	435(90.4)	358(94.7)	77(74.8)	
Death, n (%)	46(9.6)	20(5.3)	26(25.2)	
SCAP, n (%)	93(19.3)	44(11.6)	49(47.6)	<0.001
Underlying diseases				
Hypertension, n (%)	162(33.7)	129(34.1)	33(32.0)	0.726
Diabetes, n (%)	178(37.0)	134(35.4)	44(42.7)	0.205
Cardiovascular Disease, n (%)	168(34.9)	126(33.3)	42(40.8)	0.164
Chronic kidney disease, n (%)	91(18.9)	70(18.5)	21(20.4)	0.672
Cerebrovascular disease, n (%)	119(24.7)	88(23.3)	31(30.1)	0.159
COPD, n (%)	116(24.1)	87(23.0)	29(28.2)	0.299
CCI, mean (SD)	2.78(1.31)	2.56(1.26)	3.57(1.20)	<0.001
Nutritional status				<0.001
MNA-SF≥12, n (%)	120(24.9)	111(29.4)	9(8.7)	
MNA-SF8-11, n (%)	223(46.4)	187(49.5)	36(35.0)	
MNA-SF≤7, n (%)	138(28.7)	80(21.2)	58(56.3)	
Smoking, n (%)	170(35.3)	130(34.4)	40(38.8)	0.417
Polypharmacy, n (%)	158(32.8)	114(30.2)	44(42.7)	0.018
HR,beats/min, mean (SD)	79.82(9.49)	79.55(9.75)	80.82(8.48)	0.232
SBP,mmHg, mean (SD)	137.20(16.87)	137.94(17.00)	134.51(16.20)	0.068
DBP,mmHg, mean (SD)	70.34(10.10)	70.28(10.00)	70.55(10.52)	0.806
Glu,mmol/L,mean (SD)	141.65(66.65)	133.67(48.82)	170.95(104.84)	<0.001
TC,mmol/L,mean (SD)	191.72(42.40)	190.13(40.04)	197.55(49.90)	0.027
TG,mmol/L,median (IQR)	140.71(103.54,211.50)	135.84(99.12,201.77)	156.64(114.16,227.43)	0.004
AST,U/L,median (IQR)	33.00(25.00,43.00)	33.00(25.00,44.00)	35.00(27.00,44.00)	0.190
ALT,U/L,median (IQR)	27.00(20.00,37.00)	27.00(19.00,38.00)	29.00(22.00,38.00)	0.063
BUN,mmol/L,median (IQR)	20.72(15.64,28.84)	19.84(14.80,28.87)	23.52(17.92,28.84)	0.002
Scr,μmmol/L,median (IQR)	0.85(0.69,1.32)	0.85(0.69,1.33)	0.83(0.68,1.32)	0.970
ALB,g/L, mean (SD)	3.36(0.29)	3.41(0.28)	3.22(0.26)	<0.001
WBC, ×10^9^/L, mean (SD)	9.99(2.17)	9.93(2.22)	10.24(1.98)	0.200
HB,g/L, mean (SD)	126.31(18.22)	127.67(18.41)	121.33(16.64)	0.002
PLT, ×10^9^/L,mean (SD)	211.85(87.01)	213.26(80.42)	206.66(108.10)	0.495
CRP, mg/L,median (IQR)	78.20(40.40,139.60)	63.10(35.90,119.25)	143.00(69.20,197.90)	<0.001
FI-Lab,median (IQR)	0.33(0.27,0.40)	0.27(0.20,0.33)	0.40(0.33,0.53)	<0.001

Abbreviations: SCAP, Severe Community-Acquired Pneumonia; COPD, chronic obstructive pulmonary disease; CCI, charlson comorbidity index; MNA-SF, Mini Nutritional Assessment-Short Form; HR, heart rate at admission; SBP, systolic blood pressure; DBP, diastolic blood pressure; Glu, plasma glucose; TG, triglycerides; TC, total cholesterol; AST, aspartate aminotransferase; ALT, alanine aminotransferase; BUN, blood urea nitrogen; Scr, Serum creatinine; Hb Hemoglobin; PLT, platelet count; WBC, white blood cell count; CRP, C-reactive protein; ALB, serum albumin; FI-Lab, Laboratory Frailty Index; SD, standard deviation; IQR, interquartile range.

### The impact of FI-Lab on delirium

Logistic regression was used to investigate the impact of FI-Lab and MNA-SF nutritional scores on delirium. Three models were established: Model one without adjusting for confounders, Model two adjusted for age, gender, and CCI, and Model 3 further adjusted for polypharmacy, SBP, DBP, HR, WBC, HB, PLT, Scr, BUN, ALT, AST, ALB, TC, TG, Glu, and CRP. Model 1: The logistic regression analysis indicated that FI-Lab (OR = 7.095, 95% CI: 4.291–11.730) is a significant risk factor for delirium (P< 0.05). Conversely, the MNA-SF score was significantly negatively associated with delirium (OR = 0.800, 95% CI: 0.714–0.898), meaning that for each one-point increase in the MNA-SF score, the probability of delirium occurrence decreases by approximately 20%. Model 2: After adjusting for age, gender, and comorbidity index, FI-Lab remained significantly positively associated with delirium (OR = 6.758, 95% CI: 4.005–11.402), while the MNA-SF score remained negatively associated with delirium (OR = 0.847, 95% CI: 0.751–0.956), although its influence was slightly weakened compared to Model 1. Additionally, CCI was also significantly positively associated with delirium (OR = 1.597, 95% CI: 1.301–1.961). Model 3: After further adjusting for additional variables, FI-Lab remained positively associated with delirium (OR = 3.969, 95% CI: 2.123–7.419), although its effect weakened with the inclusion of more variables. The MNA-SF score became non-significant (*p* = 0.337), suggesting that when considering more variables, the predictive ability of MNA-SF for delirium decreases. Other significant variables included CCI, polypharmacy, SCAP, ALB, BUN, Glu, and CRP (Table 2).

**TABLE 2:** Logistic regression results in defferent models.

Model	Variables	β	P	OR	95%CI lower	95%CI upper
Model 1	FI-Lab	1.959	<0.001	7.095	4.291	11.730
	MNA-SF	−0.223	<0.001	0.800	0.714	0.898
Model 2	FI-Lab	1.911	<0.001	6.758	4.005	11.402
	MNA-SF	−0.166	0.007	0.847	0.751	0.956
	CCI	0.468	<0.001	1.597	1.301	1.961
Model 3	FI-Lab	1.379	<0.001	3.969	2.123	7.419
	MNA-SF	−0.071	0.337	0.931	0.805	1.077
	CCI	0.464	<0.001	1.590	1.249	2.025
	Polypharmacy	0.732	0.022	2.078	1.109	3.896
	SCAP	1.097	0.003	2.995	1.468	6.112
	ALB	−3.088	<0.001	0.046	0.012	0.180
	BUN	0.036	0.048	1.037	1.000	1.074
	Glu	0.007	0.012	1.007	1.001	1.012
	CRP	0.004	0.002	1.004	1.002	1.007

Model 1: No adjustments for covariates.

Model 2: Adjusting for sex, age, and CCI.

Model 3: Adjusting for age, sex, CCI, polypharmacy, SBP, DBP, HR, WBC, HB, PLT, scr, BUN, ALT, AST, ALB, TC, TG, glu, and CRP.

Abbreviations: FI-Lab, Laboratory Frailty Index; CCI, charlson comorbidity index; MNA-SF, Mini Nutritional Assessment-Short Form; SCAP, Severe Community-Acquired Pneumonia; HR, heart rate at admission; SBP, systolic blood pressure; DBP, diastolic blood pressure; Glu, plasma glucose; TG, triglycerides; TC, total cholesterol; AST, aspartate aminotransferase; ALT, alanine aminotransferase; BUN, blood urea nitrogen; Scr, Serum creatinine; Hb Hemoglobin; PLT, platelet count; WBC, white blood cell count; CRP, C-reactive protein; ALB, serum albumin; OR, odds ratio; CI, confidence interval.

### Prognostic value of fi-lab and combined detection

**FIGURE 2:**
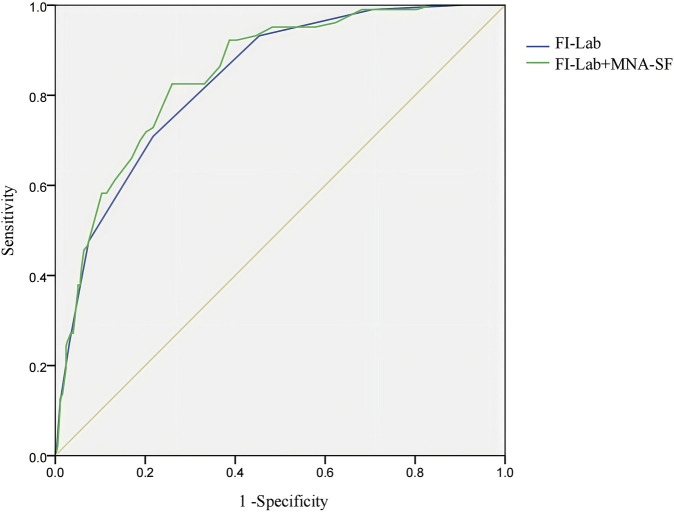
ROC curve of the predictive value Abbreviations: FI-Lab, Laboratory Frailty Index; MNA-SF, Mini Nutritional Assessment-Short Form Note: The *x*-axis represents 1 - Specificity (False Positive Rate), and the *y*-axis represents Sensitivity (True Positive Rate). Sensitivity refers to the model’s ability to correctly identify true positive cases, while specificity refers to its ability to correctly identify true negative cases.

Receiver operating characteristic (ROC) analysis assessed the sensitivity, specificity, and predictive value of the FI-Lab and its combination with MNA-SF for predicting delirium in elderly CAP patients. The AUC was 0.834 (95% CI: 0.794–0.875) for FI-Lab, with a cut-off value of 0.367 yielded 70.90% sensitivity and 78.20% specificity. Combining FI-Lab and MNA-SF increased the AUC to 0.847 (95% CI: 0.808–0 0.886), providing 82.50% sensitivity and 74.00% specificity (Figure 2).

### Kaplan-Meier survival analysis

**FIGURE 3:**
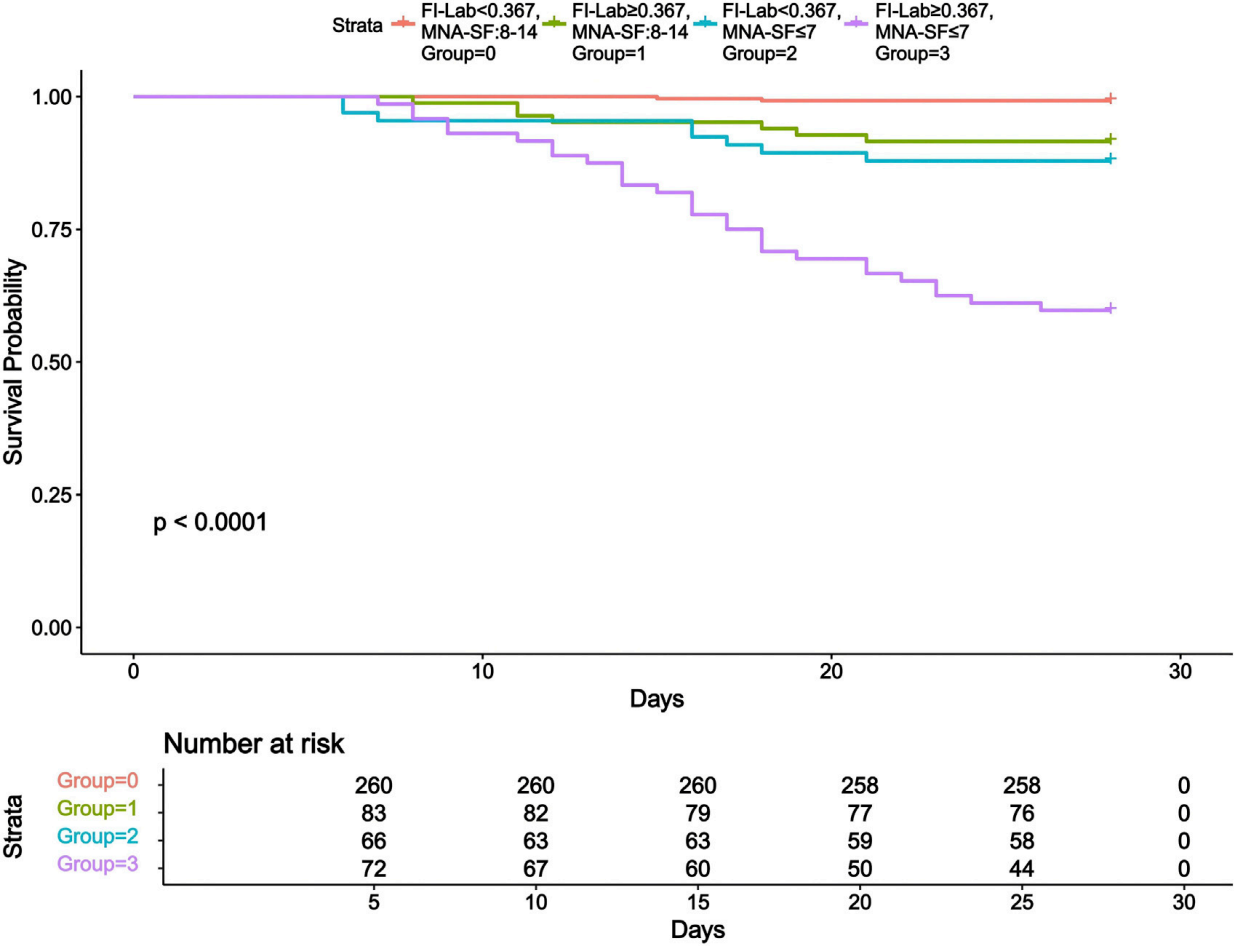
Kaplan-Meier survival curve. Abbreviations: FI-Lab, Laboratory Frailty Index; MNA-SF, Mini Nutritional Assessment-Short Form.

Based on the similarity in survival patterns and increased statistical power, we merged the MNA-SF 12-14 and MNA-SF 8–11 groups. This merging helped us more clearly demonstrate the impact of FI-Lab and nutritional status on prognosis. Further subgroup analysis revealed 28-day mortality rates of 0.8% for the group with FI-Lab < 0.367 and MNA-SF 8-14 points, 8.4% for the group with FI-Lab ≥ 0.367 and MNA-SF 8-14 points, 12.1% for the group with FI-Lab < 0.367 and MNA-SF ≤ 7 points, and 40.3% for the group with FI-Lab ≥ 0.367 and MNA-SF ≤ 7 points (Log Rank test, χ^2^ = 113.474, *p* < 0.001). Figure 3 presents the Kaplan-Meier survival curves.

### Simple mediational analyses

**FIGURE 4:**
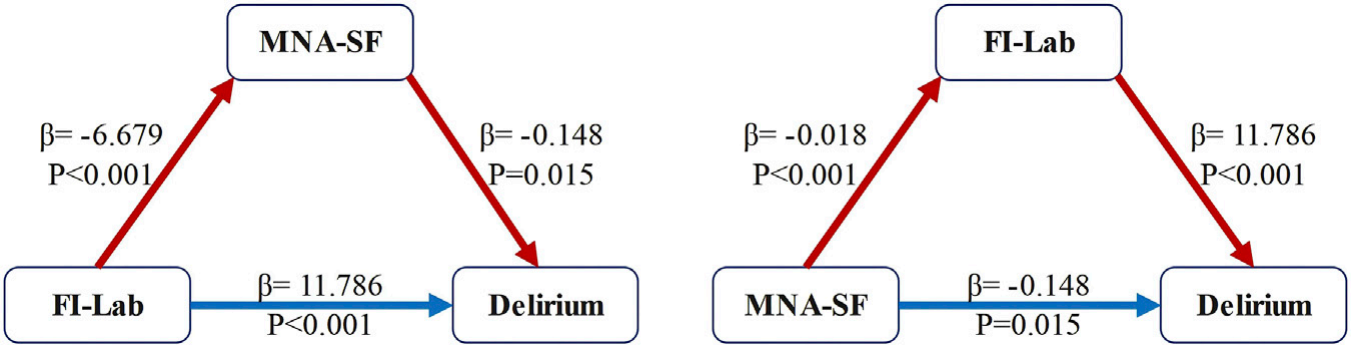
Mediation effect analysis model. Abbreviations: FI-Lab, Laboratory Frailty Index; MNA-SF, Mini Nutritional Assessment-Short Form Note: Blue solid arrows indicate the direct effects on delirium, whereas red dashed arrows denote indirect effects. Each path is labeled with its path coefficient (β) and significance level (*p*-value).

The simple mediational model, after adjusting for all potential confounding variables, revealed the following: MNA-SF mediated the relationship between FI-Lab and delirium, with an indirect effect of 0.988 (95% CI: 0.236–1.862), accounting for 7.73% of the total effect. FI-Lab also mediated the relationship between MNA-SF and delirium, with an indirect effect of −0.215 (95% CI: −0.295 to −0.153), accounting for 59.23% of the total effect. These findings suggest that FI-Lab and nutritional status not only independently serve as predictors but also interact with each other (Figure 4).

## Discussion

This study highlights the predictive value of FI-Lab for delirium in elderly patients with CAP, establishing it as a significant predictor in this population. Our results indicate that FI-Lab’s predictive accuracy is further enhanced when combined with MNA-SF. Given the complex etiology of delirium—encompassing inflammatory processes, polypharmacy, and chronic conditions—we integrated biomarkers such as C-reactive protein into the FI-Lab model, offering fresh perspectives for early identification and intervention in clinical practice.

FI-Lab provides an objective, quantitative assessment of elderly health status by analyzing multiple routine laboratory indicators (Klausen et al., 2017). Unlike traditional clinical questionnaires and physical examinations, FI-Lab minimizes assessor bias (Sapp et al., 2023). Aligning with Luo et al.'s findings, frailty significantly impacts the 30-day and 1-year mortality rates of hospitalized elderly CAP patients, affirming the frailty index’s prognostic value (Luo et al., 2020). Similarly, Kim’s research highlights FI-Lab, based on laboratory data, as an effective predictor of unfavorable outcomes like hospitalization, mortality, and long-term care needs (Kim et al., 2022). Kim et al. demonstrated that a frailty index from standard tests correlates with increased long-term mortality and institutionalization risks (Kim et al., 2019). These findings align with research by Gale et al. and Nakashima et al., which demonstrated FI-Lab’s utility in predicting adverse health outcomes and supporting clinical decision-making in elderly care (Gale et al., 2015; Nakashima et al., 2024).

Previous studies have validated FI-Lab’s predictive capabilities concerning inpatient outcomes such as readmission rates and functional decline, confirming its utility in inpatient risk assessments (Kennedy et al., 2019). Wilson et al. evaluated immunosenescence’s impact on frailty and sarcopenia, finding FI-Lab effective in predicting outcomes within these syndromes (Wilson et al., 2017). This aligns with Hakeem et al.'s findings on FI-Lab’s effectiveness in identifying high-risk patients and supporting clinical decision-making (Hakeem et al., 2023).

This study also explores the role of systemic inflammation in linking frailty, malnutrition, and delirium. Chronic low-grade inflammation, marked by elevated interleukin-6 (IL-6) and tumor necrosis factor-alpha (TNF-α), disrupts neurotransmitter balance and impairs brain function, increasing delirium risk (Parrotta et al., 2023; Wilson et al., 2017). CAP exacerbates this inflammatory response, further heightening the risk (Soysal et al., 2019). The interaction between frailty and malnutrition is significant; malnutrition can exacerbate frailty, leading to immune dysfunction and muscle loss. Conversely, frailty affects dietary habits and nutrient absorption, creating a negative cycle (Guigoz, 2006). This relationship stimulates inflammatory responses, which mediate the association between FI-Lab scores, nutritional status, and delirium risk. These findings underscore the importance of addressing both frailty and malnutrition to mitigate delirium’s impact on elderly patients.

Our research demonstrates that combining FI-Lab with MNA-SF significantly enhances predictive precision. Malnutrition is a critical factor in adverse outcomes, including delirium (Igwe et al., 2023). The MNA-SF is a valuable tool for assessing nutritional status and its association with complex health conditions. Previous studies, such as those by Zhao et al., have shown that integrating frailty and nutritional assessments improves predictions of unfavorable outcomes in elderly CAP patients (Zhao et al., 2023; da Silva et al., 2024). This study’s mediation analysis further reveals that MNA-SF mediates the relationship between FI-Lab and delirium, highlighting the intricate interplay between frailty and nutrition. These insights suggest that healthcare practices should integrate frailty and nutritional assessments to effectively predict and prevent delirium in older adults. By recognizing inflammation’s central role in connecting frailty, malnutrition, and delirium, clinicians can develop holistic management strategies. Early nutritional interventions and anti-inflammatory measures might reduce delirium cases, underscoring the necessity of utilizing both FI-Lab and MNA-SF assessments for managing elderly CAP patients.

FI-Lab offers an objective, quantitative assessment of elderly health by integrating multiple routine laboratory indicators. Unlike traditional clinical questionnaires and exams, FI-Lab minimizes bias introduced by assessor experience. However, our study has inherent limitations. As a retrospective analysis, it may be prone to selection bias due to strict inclusion criteria, potentially excluding certain patients and affecting outcome representativeness. The single-center study with a limited sample size raises concerns about generalizability. FI-Lab’s predictive capacity for delirium may vary across healthcare settings and populations. Future research should validate FI-Lab across diverse populations and centers to enhance external validity. Moreover, FI-Lab depends on multiple laboratory indicators, and variability in testing standards across hospitals could affect consistency. Standardizing laboratory protocols is crucial for reliable FI-Lab application in clinical settings. Future studies should incorporate additional biomarkers and clinical indicators to develop comprehensive predictive models, enhancing prediction accuracy and clinical applicability.

Despite the encouraging findings regarding FI-Lab’s role in predicting delirium among elderly CAP patients, a significant limitation is the lack of external validation for the FI-Lab cut-off value used. This absence may compromise generalizability. Future research should employ external cohorts to validate the FI-Lab cut-off value, improving clinical practice applicability and contributing to robust frailty assessment understanding in diverse populations.

## Conclusion

This study establishes that the FI-Lab is a robust predictor of delirium in elderly patients suffering from CAP. Its predictive accuracy is notably augmented when combined with the MNA-SF. These insights provide valuable perspectives for the early identification and intervention of delirium in clinical practice. It is recommended that healthcare providers integrate FI-Lab with nutritional assessments in managing elderly patients to enhance health outcomes.

## Data Availability

The data that support the findings of this study are not openly available due to reasons of sensitivity and de-identified data can be made available from the corresponding author upon reasonable request.

## References

[B1] Blodgett J. M. Theou O. Howlett S. E. Wu F. C. Rockwood K. (2016). A frailty index based on laboratory deficits in community-dwelling men predicted their risk of adverse health outcomes. Age Ageing 45 (4), 463–468. 10.1093/ageing/afw054 27076524

[B2] Blodgett J. M. Theou O. Howlett S. E. Rockwood K. (2017). A frailty index from common clinical and laboratory tests predicts increased risk of death across the life course. Geroscience 39 (4), 447–455. 10.1007/s11357-017-9993-7 28866737 PMC5636769

[B11] da Silva G. D. Batista A. V. D. A. Costa M. C. D. A. Dos Santos A. C. (2024). The ability of GLIM and MNA-FF to diagnose malnutrition and predict sarcopenia and frailty in hospitalized adults over 60 years of age. Front. Nutr. 11, 1456091. 10.3389/fnut.2024.1456091 39582663 PMC11583805

[B37] Dani M. Owen L. H. Jackson T. A. Rockwood K. Sampson E. L. Davis D. (2018). Delirium, frailty, and mortality: interactions in a prospective study of hospitalized older people. J. Gerontol. A Biol. Sci. Med. Sci. 73 (3), 415–418. 10.1093/gerona/glx214 29099916 PMC5861945

[B3] Ellis H. L. Wan B. Yeung M. Rather A. Mannan I. Bond C. (2020). Complementing chronic frailty assessment at hospital admission with an electronic frailty index (FI-Laboratory) comprising routine blood test results. Cmaj. 192 (1), E3–E8. 10.1503/cmaj.190952 31907228 PMC6944299

[B5] Fan J. Yu C. Guo Y. Bian Z. Sun Z. Yang L. (2020). Frailty index and all-cause and cause-specific mortality in Chinese adults: a prospective cohort study. Lancet Public Health 5 (12), e650–e660. 10.1016/S2468-2667(20)30113-4 33271078 PMC7708389

[B6] Gale C. R. Cooper C. Sayer A. A. (2015). Prevalence of frailty and disability: findings from the English longitudinal study of ageing. Age Ageing 44 (1), 162–165. 10.1093/ageing/afu148 25313241 PMC4311180

[B7] Green J. R. Smith J. Teale E. Collinson M. Avidan M. S. Schmitt E. M. (2019). Use of the confusion assessment method in multicentre delirium trials: training and standardisation. BMC Geriatr. 19 (1), 107. 10.1186/s12877-019-1129-8 30991945 PMC6466721

[B15] Guan L. Soh C. H. Reijnierse E. M. Maier A. B. (2022). Association of a modified laboratory frailty index with adverse outcomes in geriatric rehabilitation inpatients: RESORT. Mech. Ageing. Dev. 203, 111648. 10.1016/j.mad.2022.111648 35219637

[B8] Guigoz Y. (2006). The Mini Nutritional Assessment (MNA) review of the literature—what does it tell us? J. Nutr. Health Aging 10 (6), 466–485. 17183419

[B35] Hakeem F. F. Maharani A. Todd C. O'Neill T. W. (2023). Development, validation and performance of laboratory frailty indices: a scoping review. Arch. Gerontol. Geriatr. 111, 104995. 10.1016/j.archger.2023.104995 36963345

[B9] Ide K. Banno T. Yamato Y. Hasegawa T. Yoshida G. Yasuda T. (2021). Relationship between locomotive syndrome, frailty and sarcopenia: locomotive syndrome overlapped in the majority of frailty and sarcopenia patients. Geriatr. Gerontol. Int. 21 (6), 458–464. 10.1111/ggi.14162 33825291

[B20] IgweE. O.DingP.CharltonK. E.NealonJ.TraynorV. (2023). Association between malnutrition and delirium in older chronic kidney disease patients admitted to intensive care units: A data linkage study. J. Nutr. Health 27 (7), 571–577. 10.1007/s12603-023-1938-5 37498104

[B10] Inouye S. K. Westendorp R. G. Saczynski J. S. (2014). Delirium in elderly people. The Lancet 383 (9920), 911–922. 10.1016/S0140-6736(13)60688-1 PMC4120864 23992774

[B12] Kennedy C. C. Novotny P. J. LeBrasseur N. K. Wise R. A. Sciurba F. C. Benzo R. P. (2019). Frailty and clinical outcomes in chronic obstructive pulmonary disease. Ann. Am. Thorac. Soc. 16 (2), 217–224. 10.1513/AnnalsATS.201803-175OC 30433830 PMC6376949

[B14] Kim D. J. Massa M. S. Potter C. M. Clarke R. Bennett D. A. (2022). Systematic review of the utility of the frailty index and frailty phenotype to predict all-cause mortality in older people. Syst. Rev. 11 (1), 187. 10.1186/s13643-022-02052-w 36056441 PMC9438224

[B26] Kim Y. P. Choe Y. R. Park J. H. Kim S. Won C. W. Hwang H. S. (2019). Frailty index associated with all-cause mortality, long-term institutionalization, and hip fracture. Eur. Geriatr. Med. 10 (3), 403–411. 10.1007/s41999-019-00196-y 34652803

[B17] Klausen H. H. Petersen J. Bandholm T. Juul-Larsen H. G. Tavenier J. Eugen-Olsen J. (2017). Association between routine laboratory tests and long-term mortality among acutely admitted older medical patients: a cohort study. BMC Geriatr. 17 (1), 1–14. 10.1186/s12877-017-0434-3 28249621 PMC5333426

[B39] Liu H. Jiao J. Zhu M. Wen X. Jin J. Wang H. (2022). Nutritional status according to the short-form mini nutritional assessment (MNA-SF) and clinical characteristics as predictors of length of stay, mortality, and readmissions among older inpatients in China: a national study. Front. Nutr. 9, 815578. 10.3389/fnut.2022.815578 35145987 PMC8822231

[B4] Luo J. Tang W. Sun Y. Jiang C. (2020). Impact of frailty on 30-day and 1-year mortality in hospitalised elderly patients with community-acquired pneumonia: a prospective observational study. BMJ open 10 (10), e038370. 10.1136/bmjopen-2020-038370 PMC7783614 33130565

[B18] Marcantonio E. R. (2017). Delirium in hospitalized older adults. N. Engl. J. Med. 377 (15), 1456–1466. 10.1056/NEJMcp1605501 29020579 PMC5706782

[B19] Metlay J. P. Waterer G. W. Long A. C. Anzueto A. Brozek J. Crothers K. (2019). Diagnosis and treatment of adults with community-acquired pneumonia. An official clinical practice guideline of the American Thoracic Society and Infectious Diseases Society of America. Am. J. Respir. Crit. Care Med. 200 (7), e45–e67. 10.1164/rccm.201908-1581ST 31573350 PMC6812437

[B16] Moreno C. Mueller S. Szabo G. (2019). Non-invasive diagnosis and biomarkers in alcohol-related liver disease. J. Hepatol. 70 (2), 273–283. 10.1016/j.jhep.2018.11.025 30658728

[B13] Nakashima H. Watanabe K. Komiya H. Fujisawa C. Yamada Y. Sakai, T. (2024). Frailty index based on common laboratory tests for patients starting home-based medical care. J. Am. Med. Dir. Assoc. 25 (9), 105114 10.1016/j.jamda.2024.105114 38950586

[B23] Parrotta I. Bencivenga L. Okoye C. Bellelli G. Fumagalli S. Mossello E. (2023). Frailty and hyperactive delirium in hospitalized older patients with COVID-19: an insight from GeroCovid registry. Aging Clin. Exp. Res. 35 (2), 433–442. 10.1007/s40520-022-02328-0 36600029 PMC9812736

[B21] Patel K. V. (2008). Epidemiology of anemia in older adults. Semin. Hematol. 45 (4), 210–217. 10.1053/j.seminhematol.2008.06.006 18809090 PMC2572827

[B22] Ridker P. M. (2014). Inflammation, C-reactive protein, and cardiovascular disease: moving past the marker versus mediator debate. Circ. Res. 114 (4), 594–595. 10.1161/CIRCRESAHA.114.303215 24526672

[B38] Resendes N. M. Chada A. Torres-Morales A. Fernandez M. Diaz-Quiñones A. Gomez C. (2023). Association between a frailty index from common laboratory values and vital signs (FI-LAB) and hospital and post-hospital outcomes in veterans with COVID-19 infection. J. Nutr. Heal. Aging. 27 (2), 89–95. 10.1007/s12603-023-1886-0 PMC9893965 36806863

[B24] Rockwood K. McMillan M. Mitnitski A. Howlett S. E. (2015). A frailty index based on common laboratory tests in comparison with a clinical frailty index for older adults in long-term care facilities. J. Am. Med. Dir. Assoc. 16 (10), 842–847. 10.1016/j.jamda.2015.03.027 25952475

[B25] Rockwood K. Mitnitski A. B. (2007). Frailty in relation to the accumulation of deficits. J. Gerontol. A Biol. Sci. Med. Sci. 62 (7), 722–727. 10.1093/gerona/62.7.722 17634318

[B27] Sahle B. W. Pilcher D. Litton E. Ofori-Asenso R. Peter K. McFadyen J. (2022). Association between frailty, delirium, and mortality in older critically ill patients: a binational registry study. Ann. Intensive Care 12 (1), 108. 10.1186/s13613-022-01080-y 36394660 PMC9672151

[B28] Sanchez D. Brennan K. Al Sayfe M. Shunker S. A. Bogdanoski T. Hedges S. (2020). Frailty, delirium and hospital mortality of older adults admitted to intensive care: the Delirium (Deli) in ICU study. Crit. Care 24, 609. 10.1186/s13054-020-03318-2 33059749 PMC7565834

[B29] Sapp D. G. Cormier B. M. Rockwood K. Howlett S. E. Heinze S. S. (2023). The frailty index based on laboratory test data as a tool to investigate the impact of frailty on health outcomes: a systematic review and meta-analysis. Age Ageing 52 (1), afac309. 10.1093/ageing/afac309 36626319 PMC9831271

[B30] Soysal P. Veronese N. Arik F. Kalan U. Smith L. Isik A. T. (2019). Mini Nutritional Assessment Scale-Short Form can be useful for frailty screening in older adults. Clin. Interv. Aging 14, 693–699. 10.2147/CIA.S196770 31118593 PMC6475097

[B31] Torres A. Chalmers J. D. Dela Cruz C. S. Dominedò C. Kollef M. Martin-Loeches I. (2019). Challenges in severe community-acquired pneumonia:a point-of-view review. Intensive Care Med. 45, 159–171. 10.1007/s00134-019-05519-y 30706119 PMC7094947

[B32] Wang C. Fang X. Tang Z. Hua Y. Zhang Z. Gu X. (2022). A frailty index based on routine laboratory data predicts increased risk of mortality in Chinese community-dwelling adults aged over 55 years: a five-year prospective study. BMC Geriatr. 22 (1), 679. 10.1186/s12877-022-03374-z 35978284 PMC9382755

[B33] Williams B. Mancia G. Spiering W. Agabiti Rosei E. Azizi M. Burnier M. (2018). 2018 ESC/ESH Guidelines for the management of arterial hypertension. Eur. Heart J. 39 (33), 3021–3104. 10.1093/eurheartj/ehy339 30165516

[B36] Wilson D. Jackson T. Sapey E. Lord J. M. (2017). Frailty and sarcopenia: the potential role of an aged immune system. Ageing Res. Rev. 36, 1–10. 10.1016/j.arr.2017.01.006 28223244

[B34] Zhao L. H. Chen J. Zhu R. X. (2023). The relationship between frailty and community-acquired pneumonia in older patients. Aging Clin. Exp. Res. 35 (2), 349–355. 10.1007/s40520-022-02301-x 36447006

